# Foreword: Public health, public policy, politics and policing

**DOI:** 10.1186/1477-7517-9-22

**Published:** 2012-07-09

**Authors:** Daniel Tarantola

**Affiliations:** 1 Visiting Professorial Fellow, School of Public Health and Community Medicine, Faculty of Medicine, The University of New South Wales, Sydney, NSW 2052, Australia

## Abstract

Reducing harm from drug use lies at the intersection of public health, public policy, politics and policing. In an ideal world, evidence of public health gains achievable through new approaches or technologies should inform public policy, should help shape political agendas in support of policy change, which should translate into law and regulations – and then to their application. The goal of this transformative process should be to yield the highest attainable health benefits to vulnerable individuals and communities and to society as a whole.

## 

Harm reduction, as a public health, encompasses a wide and diverse array of approaches aimed at minimizing individual and collective negative impacts of risky behaviours. Road safety measures such as speed limitation and the enforcement of seat belt and motorcycle helmet legislation are examples of such measures. In the realm of HIV/AIDS, harm reduction approaches have had significant impacts on HIV transmission among men who have sex with men (MSM) and injecting drug users (IDUs) as well as in the context of heterosexual and transactional sex. Elimination of risk, vulnerability and impact will remain remote goals until a cure and highly effective vaccines become available, and most-at-risk populations are no longer subjected to discrimination and social exclusion. Until then, harm reduction approaches have proven the approaches of choice, demonstrated by declining HIV prevalence and incidence over the last two decades in populations where prevention and early access to treatment have been instituted at scale.

Evidence abounds supporting the introduction in public policy and in practice of measures aimed principally at minimizing the spread of HIV (and Hepatitis B and C) resulting from the sharing of unsterile paraphernalia. Those who have patiently and rigorously collected, analysed and disseminated this evidence have produced a very robust body of evidence for policy change. Often in adverse environments, they have established that making sterile injection equipment available to drug users and switching from injection – involving high risks of blood-borne contamination – to less harmful forms of drug use, including the oral administration of opiate substitutes, substantially lowers risk and transmission of HIV. They have repeatedly shown that these measures are effective on the individual and community levels when combined with individual counselling, community networking and public information. Notably, they have also documented that these approaches do not impact negatively on public health, for example by fuelling the widespread use of drugs in non-user populations. Funding agencies that have supported this work deserve special recognition too.

Thus, the evidence strongly militates in favour of structural changes that lower vulnerability to HIV and other blood-borne infections, by including drug-related harm reduction in all prevention programmes targeted at IDUs. Yet desirable policy changes have been hampered by ignorance, neglect or denial within government and political circles. As Albert Camus put it: “*By definition, a government has no conscience. Sometimes it has a policy, but nothing more.”* And even where sound policies exist, they are not sufficiently or not at all supported by legislation, or even less so by state capacity to enforce it. Informed by evidence, political agendas should be geared to induce public understanding and acceptance of these changes and support their entrenchment in existing or new legislation and regulations. Regrettably, however, a disconnect remains between such policies as may be embodied in national HIV/AIDS strategies, on the one hand, and laws decrees and regulations that prohibit its implementation, on the other.

In turn, when the legislation allows, law enforcement should recognize and respect the newly established boundaries within which they are set to operate. These boundaries are however blurred, and the interpretation and enforcement of the law inconsistent. The seemingly logical chain linking public health rationale to policy, politics and policing has many weak links and is divorced from reality in most countries, regardless of their level of economic development. Politicians have not displayed the leadership and pragmatism needed to introduce reforms perceived as benefiting marginalized populations primarily treated as law offenders or even labelled as the manifestation of “social evils”.

Paradoxically – in every sense of the term – law enforcement authorities have occasionally displayed greater understanding than policy makers and legislators that neither the “war on drugs” nor HIV prevention are served well by occasional, random crack-downs on drug users or their deprivation of access to harm reduction methods. Where ineffective laws disallow any interaction other than repression and coercion between enforcement officers and drug users, the interpretation of the law is left to those expected to enforce it. Such interpretation is dangerously flawed, however, by the lack of clarity about what comprehensive drug-related harm reduction should actually entail in given geopolitical and epidemiological contexts, the inadequacy of guidance and skills received by law enforcement officers, the misconceived incentives offered them to stimulate their interventions, and abuses of power often associated with personal profits.

Ever since HIV emerged, policing has been blamed for creating obstacles to access by affected communities to prevention programmes primarily intended for their benefit. Yet, in almost all Southeast Asian nations, ground-breaking harm reduction projects have been tolerated even though they were not entrenched in, or were prohibited by policies and laws. The tolerance which benefited a number of these projects, as fragile, unpredictable and subjected to repeated setbacks as it was, has allowed projects to gather the evidence supporting harm reduction approaches, and, combined with international advocacy, to persuade policy makers that it was time for a change.

Drawing from selected Southeast Asian country case-studies, this very timely Special Issue takes a pragmatic look at opportunities and barriers to effect this change. Specifically, it examines some of the factors that have determined the conflictual relationship between law enforcement and the protection of public health. It suggests that policing, when well conceived and implemented, actually constitutes a largely untapped resource in HIV prevention benefiting substance users and the rest of the population. As several articles in this Issue underscore, progress in this direction does not merely imply sensitizing law enforcement services to sound public health practices by imposing on them a public health agenda laid out in public health terms they may not be familiar with. It also implies recasting and supporting the role of these services taking into account their obligations, structures, competing priorities, accountability, cultural specificity and the social context within which they operate. Several of these factors of change, as well as the required caution and the associated risks in acting on them, are invoked in this excellent collection of articles, in the hope of constructing a new understanding of how policing must contribute, rather than hamper, the prevention of HIV transmission through IDU. Borrowing from Jawaharlal Nehru: “The policy of being too cautious is the greatest risk of all.”

